# Glacial Lake Area Changes in High Mountain Asia during 1990–2020 Using Satellite Remote Sensing

**DOI:** 10.34133/2022/9821275

**Published:** 2022-10-21

**Authors:** Meimei Zhang, Fang Chen, Huadong Guo, Lu Yi, Jiangyuan Zeng, Bin Li

**Affiliations:** ^1^International Research Center of Big Data for Sustainable Development Goals, Beijing 100094, China; ^2^Key Laboratory of Digital Earth Science, Aerospace Information Research Institute, Chinese Academy of Sciences, No. 9 Dengzhuang South Road, Beijing 100094, China; ^3^University of Chinese Academy of Sciences, Beijing 100049, China; ^4^Key Laboratory of Coastal Environment and Resources Research of Zhejiang Province, School of Engineering, Westlake University, Hangzhou 310024, China; ^5^State Key Laboratory of Remote Sensing Science, Aerospace Information Research Institute, Chinese Academy of Sciences, Beijing 100094, China

## Abstract

Changes in a large-scale glacial lake area directly reflect the regional glacier status and climate changes. However, long time series of glacial lake dataset and comprehensive investigation of the spatiotemporal changes in the glacial lake area in the whole High Mountain Asia (HMA) region remained elusive. Satellite remote sensing provides an indispensable way for dynamic monitoring of glacial lakes over large regions. But glacial lakes are quite small and discretely distributed, and the extraction of glacial lakes is usually influenced by clouds, snow/ice cover, and terrain shadows; thus, there is a lack of an automatic method to continuously monitor the dynamic changes of glacial lakes in a large scale. In this paper, we developed a per-pixel composited method named the “multitemporal mean NDWI composite” to automatically extract the glacial lake area in HMA from 1990 to 2020 using time-series Landsat data. There were 19,294 glacial lakes covering a total area of 1471.85 ± 366.42 km^2^ in 1990, and 22,646 glacial lakes with an area of 1729.08 ± 461.31 km^2^ in 2020. It is noted that the glacial lake area in the whole HMA region expanded by 0.58 ± 0.21%/a over the past three decades, with high spatiotemporal heterogeneity. The glacial lake area increased at a consistent speed over time. The fastest expansion was in East Kun Lun at an average rate of 2.01 ± 0.54%/a, while in the Pamir and Hengduan Shan, they show slow increases with rates of 0.33 ± 0.08%/a and 0.39 ± 0.01%/a, respectively, during 1990–2020. The greatest increase in lake area occurred at 5000-5200 m a.s.l., which increased by about 45 km^2^ (~25%). We conclude that the temperature rise and glacier thinning are the leading factors of glacial lake expansion in HMA, and precipitation is the main source of lake water increase in West Kun Lun. Using the proposed method, a large amount of Landsat images from successive years of melting seasons can be fully utilized to obtain a pixel-level composited cloud-free and solid snow/ice-free glacial lake map. The uncertainties from supraglacial ponds and glacial meltwater were also estimated to improve the reliability and comparability of glacial lake area changes among different regions. This study provides important technical and data support for regional climate changes, glacier hydrology, and disaster analysis.

## 1. Introduction

Glacial lakes are the joint product of global warming and glacier melting. Glacial lake changes are closely associated with climate changes and glacier activities and impact the local hydrologic cycles [1, 2]. High Mountain Asia (HMA) develops the most extensive glaciers in the middle and low latitudes [3, 4]. In addition to evaporation and flowing into rivers, a fair amount of glacial meltwater is retained in the glacial lakes. This to some extent delays the loss of regional glacial water resources due to climatic warming [5, 6] but also directly leads to an increased risk and number of potentially dangerous glacial lakes [7]. HMA is known as the world's Glacial Lake Outburst Flood (GLOF) vulnerability hotspot [1, 8], and its GLOF risk could nearly triple with the future rapid development of lakes [9]. In the periods of 1990 and 2018, it was estimated that the HMA glacial lakes experienced widespread areal expansion at an average rate of 15.2% [10], with large variability between different subregions and elevation ranges.

Glacial lake area extraction is basically important for two reasons. First, glacial lake coverage areas offer the basic data to determine the detailed lake outlines. Several recently released HMA glacial lake inventories [10, 11], which are mainly delineated manually from Landsat images for the boundaries, can be used to provide the fundamental data for glacial lake change detection, GLOF hazard analysis, and terrestrial water budgets. Second, glacial lake coverage and associated area changes are closely related to glacier mass balance changes and regional climate conditions. Simulation results showed that more than 13,000 new glacial lakes could appear in the nonglacial area, and ~47% of them will appear by 2050 [9]. However, for all HMA glacial lakes, there are few long-term, continuous, and systematic observations of area changes so far. Most of the efforts are concerned with glacial lake monitoring over different regions of HMA at different time scales [12–14]. The resulting incomplete spatial coverage and the influence of different data sources and mapping algorithms yield large uncertainties in the regional comparisons and comprehensive evaluation of HMA [6, 15].

In the past decades, many studies have been carried out to delineate glacial lakes using optical remote sensing images. Manual digitalization through visual interpretation is still a necessary step for optimizing the mapping results. Water indices (WIs) [16–18] combine two or more spectral bands by mathematical computation and are effective and convenient for water detection. The combination of WI maps with several segmentation methods, such as iterative threshold segmentations [19–21] and level-set-based active-contour approaches [15, 22], has been proposed to improve the lake local details. Nevertheless, a number of manual postprocessing steps were required to remove other false water features (e.g., small streams and shadows) and supplement missing lakes. Object-oriented analysis of remote sensing data that included size, shape, reflectance, and contextual information was performed for mapping glacial lakes with specific morphological and spectral features [21, 23]. Machine learning, such as random forest and deep learning methods, has high generalization and automation ability for recognizing glacial lakes from high-dimensional data, while the results heavily rely on the appropriate training samples. Additionally, the requirements for both costs and processing times are exceptionally high [24–26]. Despite the achieved inspiring results, for HMA, the extraction of small and dispersed glacial lake targets over such a large-scale area with harsh climate and complex terrain conditions is challenging. Among the aforementioned methods, WIs are the most popular method for glacial lake extraction due to their high computational efficiency and ease of use, and among different WIs, the normalized difference water index (NDWI) [16] is robust in delineating different types of lakes and features a reasonably stable threshold to classify edge pixels of water [18, 27].

Glacial lakes are defined as water bodies originating from glacier activities, which are formed when glaciers erode the lands and melt and fill depressions excavated by glaciers [28]. According to the definition of glacial lakes, they are distributed around glaciers, and a 10 km buffer [11, 29] around the Randolph Glacier Inventory (RGI v6.0) [30] was widely used to preliminarily determine the distribution area of glacial lakes. Within this 10 km buffer range, some researchers only define the lakes that are directly fed by glaciers as glacial lakes [31, 32], but others think that all the lakes within the buffer are glacial lakes and can be further divided into different subclasses according to whether they are supplied by glaciers and whether they are connected with glaciers [10, 33]. There are no unified classification schemes for glacial lakes. Unlike glaciers that appear as perennial, large pieces of dense ice slowly moving under their own weight, glacial lakes are abundant at small size and have high spatial variability. Due to the changeable environment and the complex spectral features of satellite images, the great challenges of automatically detecting glacial lakes from optical data include the following obstacles: identifying clouds and alleviating their impacts, excluding mountain shadows, distinguishing glacial lakes from snow and ice cover, and detecting the changing pattern of glacial lakes.

Summer and autumn seasons are generally selected as the optimal time to recognize glacial lakes from satellite imagery because ice and seasonal snow melt and lakes present a stable extent during this period [34]. For the whole HMA, the situation is much more complicated. There is frequent solid precipitation in summer in the Himalayan regions [35], and this is especially pronounced on Tien Shan Mountain, where the precipitation is solid for the whole year at an elevation higher than 3000 m [36, 37]. The existence of clouds and seasonal snow cover in the warm season makes lake delineation challenging. Glacial lake inventory compilation for the southeastern Tibetan Plateau is extremely difficult due to the prevalent heavy clouds influenced by summer monsoons and high-relief terrain [38]. Although clouds and cloud shadows can be automatically detected from multispectral data and using time series of satellite scenes could reduce the impact of clouds, it is not possible to merge them together piecewise for large cloud-free optical imagery.

In rugged terrain environments, glacial lakes are easily confused with mountain shadows due to their similar spectral reflectance. This phenomenon is particularly serious for north-facing glaciers that are widely shaded by adjacent mountains [39]. Slope maps and shaded relief derived from a digital elevation model (DEM) have been employed to mask a number of mountain shadows [9, 22]. To completely eliminate the interference of mountain shadows, their spatial relationships with regard to glacial lakes should be modeled, which requires many additional parameters such as solar elevation angle, local time of image, and relative height difference between glacial lakes and mountain peaks [40]. It is not easy to identify mountain shadows in large regions.

At present, there is no systematic studies to investigate the current distribution and spatiotemporal heterogeneity of glacial lakes in the whole HMA. The historical to present glacial lake dataset covering HMA is crucial for assessing glacier-climate change interactions and GLOF risk at the regional scale. Local residents and management agencies are also very concerned about the development and changes of glacial lakes, in order to predict and give early warning of outburst disasters and minimize casualties and economic losses. Meanwhile, monitoring glacial lake changes in HMA as a whole is particularly important for assessing the impacts of global climate changes. The spatial distribution and heterogeneous changes of rapidly expanding glacial lakes in the whole HMA region deserve further attentions.

Google Earth Engine (GEE) is the most advanced geographic information processing platform based on cloud computing in the world. It offers the possibility for the large-scale processing and analysis of geoscience data and has become popular in land-cover classification [41–43]. For mapping glacial lakes over the whole HMA region, a reasonable and effective strategy is still lacking. In the study of the extraction of glacial lake outlines using this cloud-based platform, the scheme of glacial lake mapping in the Third Pole is discussed [22]. However, the GEE is mainly used for the selection of cloudless images, and we noted the postprocessing problems regarding manual reediting and removing some erroneously extracted lakes in their semiautomated method. Therefore, in this study, we developed a new automatic method—the multitemporal mean NDWI composite—to extract glacial lakes based on the archived Landsat data on GEE. Then, the glacial lake coverage over the whole HMA was extracted with the proposed method, and long-term changes in the glacial lake area from 1990 to 2020 were estimated in detail.

## 2. Results

### 2.1. Spatial Distribution Pattern of Glacial Lakes

**Figure 1:**
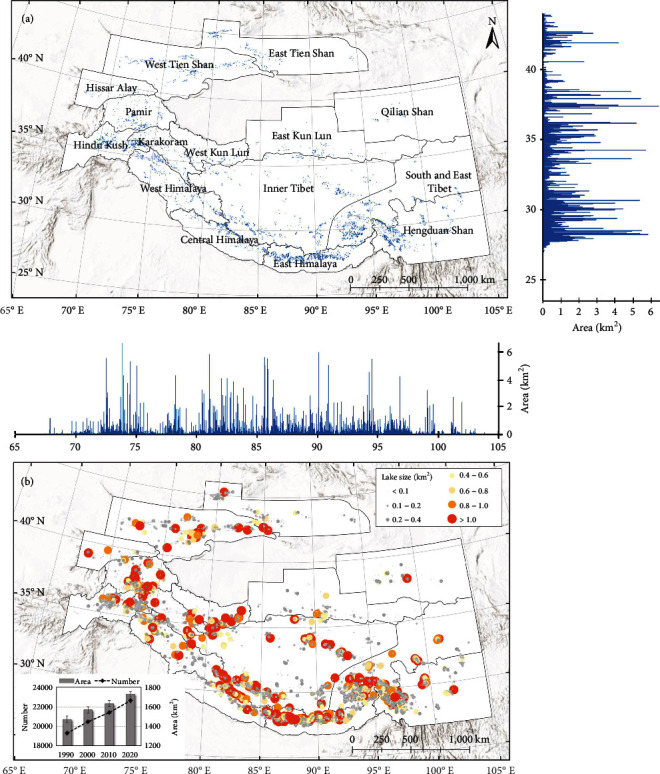
Glacial lake distribution in HMA in 2020. (a) The mapped glacial lake extent (blue polygons), and area statistics along latitude (right) and longitude (below); (b) distribution of different sizes of glacial lakes. The circle size represents the size class to which each glacial lake area belongs. The inset shows the total number and area of glacial lakes in 1990, 2000, 2010, and 2020.

Based on the developed glacial lake mapping method, the glacial lake areas across the 15 subregions in HMA were extracted at 10-year intervals from 1990 to 2020. An example of the latest distribution pattern of glacial lakes in 2020 is presented in Figure 1. Most of the glacial lakes were developed in the southern zone of HMA, including Hindu Kush-Karakoram-Himalaya Mountain ranges, South and East Tibet, and Hengduan Shan (Figure 1(a)), where large glacial lakes (>1 km^2^) were also distributed over these regions (Figure 1(b)). In East Kun Lun and Qilian Shan, glacial lakes were sparsely distributed, and almost all were in small sizes. Glacial lake area is evenly distributed along the longitude, but concentrated in the latitude range between 27°N and 32°N and also around 35°N, corresponding to the Central and East Himalaya, the south side of South and East Tibet, and the Hengduan Shan (Figure 1(a)). In 2020, only 214 large glacial lakes (>1 km^2^) were identified. Glacial lakes with an area of less than 1 km^2^ covered 1337.36 ± 261.47 km^2^, accounting for 77.35 ± 18.16% of the total lake area (1729.08 ± 461.31 km^2^). 19,131 glacial lakes (578.37 ± 121.45 km^2^) are less than 0.1 km^2^, which is dominated in number (84%). Compared with the very large glacial lakes, the spatial and temporal variation of these small lakes is significant due to the unstable shapes and enhanced hydrological cycle [21].

**Figure 2:**
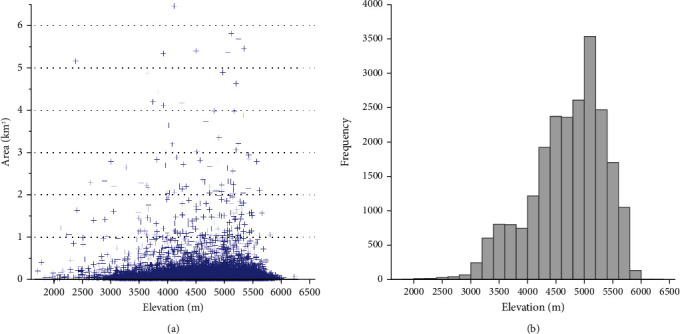
(a) Area distribution of glacial lakes along the elevation in 2020; (b) frequency distribution of glacial lakes along the elevation.

Figure 2 shows the distribution pattern of glacial lakes in the vertical direction in 2020. Glacial lakes were distributed between 2000 m a.s.l. and 6000 m a.s.l. Most of the glacial lakes were located at the elevation above 3500 m a.s.l., and the main elevation range of the glacial lake distribution was 4200–5500 m a.s.l. These results are consistent well with the previous studies [10, 11, 31]. In each 200 m bin size, more than 3500 glacial lakes were found in the 5000–5200 m a.s.l., which further confirm that as the global temperature rises, more glacial lakes gradually develop towards higher elevations [44].

### 2.2. Area Changes in Glacial Lakes in HMA

**Figure 3:**
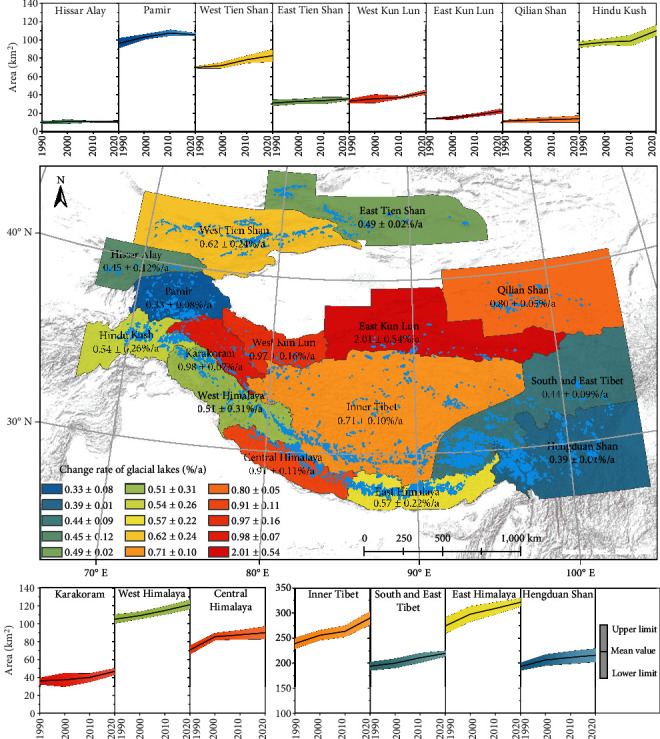
Glacial lake area changes in HMA during 1990–2020. Map in the center displays the spatial extent of HMA, glacial lake distribution (blue polygons) in 2020, and the average change rate in each subregion during 1990–2020. Banded curves in different colors denote the detailed glacial lake area changes in different periods and different subregions.

Due to the occurrence of snow or ice meltwater on the glacier terminus and the limited observation times of each pixel used for the image composite in some regions, the extracted glacial lake pixels cannot be directly used for the calculation and analysis of glacial lake area changes. To reduce the total bias in the estimated lake area and improve the reliability of results, lake area uncertainty is estimated by an error of areas corresponding to the lake region within the glacier boundaries. Our mapped glacial lake pixels corresponding to the region outside of glacier boundaries are used as the lower limit, and all glacial lake pixels are deemed the upper limit. The areas within the upper and lower limits in different colors in Figure 3 denote the glacial lake area changes of each subregion during 1990–2020. Considering the presence of unstable supraglacial lakes on the glacier surface, the estimated glacial lake area of each subregion is obtained by averaging the upper and lower limits.

We do not adopt the uncertainty estimation methods used in the previous studies by the linear error and the lake's perimeter [39, 45, 46]. The main reasons are as follows: (1) the proposed automated mapping method is based on the pixel-level composited, and the output glacial lake maps are in raster format displaying the glacial lake coverage areas, while others are vectorized glacial lake outlines; (2) the effects from supraglacial ponds and glacial meltwater and the limited observation times are fully considered in this study. Based on the calculated glacial lake areas and their uncertainty measurements, the area change rates between 1990 and 2020 in different regions are calculated.

The lake area change with time varies greatly among different regions across the HMA. All the 15 subregions showed varying degrees of expansion. This is expected due to the continued global warming (especially in the HMA region) over the past several decades [47, 48]. The most rapid expansion in the glacial lake area is in East Kun Lun at a rate of approximately 2.01 ± 0.54%/a, but with a small total area (22.30 ± 4.36 km^2^ in 2020). There has been little expansion in Hengduan Shan during the past 30 years. In Pamir, the glacial lake areas increased rapidly during 1990–2010, but slightly decreased and became relatively stable in the following 10 years, resulting in the slowest expansion rate (0.33 ± 0.08%/a). It should be noted that in West Kun Lun, where the glacier area was recorded to increase at a rate of 0.50 ± 0.11%/a during 1990–2018 [49], glacial lakes in this region also unexpectedly exhibited an increasing area in recent years.

**Figure 4:**
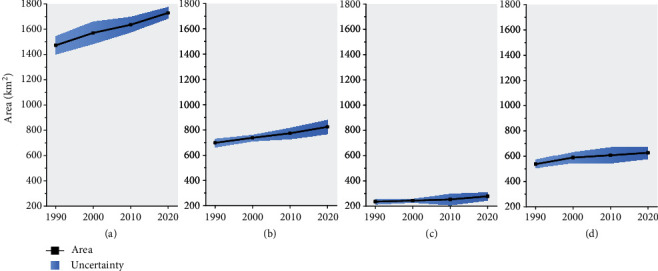
Glacial lake area changes from 1990 to 2020 for (a) the whole HMA, (b) Central Asia (including Hissar Alay, Pamir, West Tien Shan, East Tien Shan, West Kun Lun, East Kun Lun, Qilian Shan, Inner Tibet, and South and East Tibet), (c) South Asia West (including Hindu Kush, Karakoram, and West Himalaya), and (d) South Asia East (including Central Himalaya, East Himalaya, and Hengduan Shan).

The changes in the glacial lake area in the whole HMA and Region 13 Central Asia, Region 14 South Asia West, and Region 15 South Asia East are shown in Figure 4. Glacial lakes expanded at rates of 0.60 ± 0.17%/a, 0.59 ± 0.23%/a, and 0.55 ± 0.11%/a between 1990 and 2020 for Central Asia, South Asia West, and South Asia East, respectively. The total glacial lake area in HMA expanded by 257.23 ± 94.89 km^2^ (17.47 ± 6.33%) at a rate of 0.58 ± 0.21%/a during 1990–2020. Specifically, the expansion rates of glacial lake area were 0.67 ± 0.12%/a from 1990 to 2000, 0.40 ± 0.05%/a from 2000 to 2010, and 0.57 ± 0.09%/a from 2010 to 2020, with a continuous and uniformly expanding trend in the past 30 years.

### 2.3. Different Patterns of Glacial Lake Evolution

**Figure 5:**
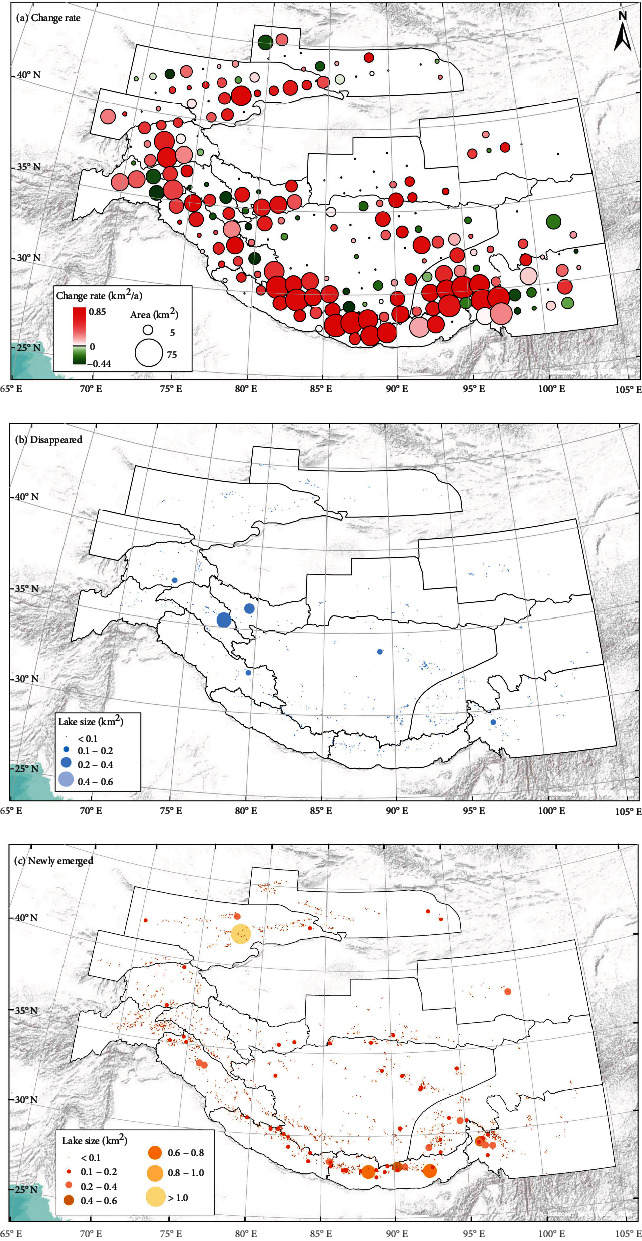
Evolution pattern of glacial lakes in HMA from 1990 to 2020. (a) Change rates in glacial lake area aggregated on a 1° × 1° grid. The circle size represents the total glacial lake area in 2020. (b) Disappeared glacial lakes. (c) Newly emerged glacial lakes.

In order to explore the dynamic evolution process of glacial lakes in more detail, the annual area changes of glacial lakes aggregated on a grid of 1° × 1° were further analyzed (Figure 5(a)). Negative growth of glacial lake area was observed in East Hindu Kush, Karakoram, Inner Tibet, and Hengduan Shan. The region with the fastest area decrease was in the Karakoram Mountain, with a negative area rate of -0.44 km^2^/a, where a medium-sized glacial lake (0.4-0.6 km^2^) had vanished during 1990-2020 (Figure 5(b)). In contrast, the areas of glacial lakes in most of the other mountains continue to expand. The fastest growing region (0.85 km^2^/a) is located in the East Himalaya, with the emergence of many medium and large glacial lakes in 2020 (Figure 5(c)). It is noted that a new glacial lake with an area of larger than 1 km^2^ has appeared in the West Tien Shan, which also correspond with the rapidly expanding zone.

**Figure 6:**
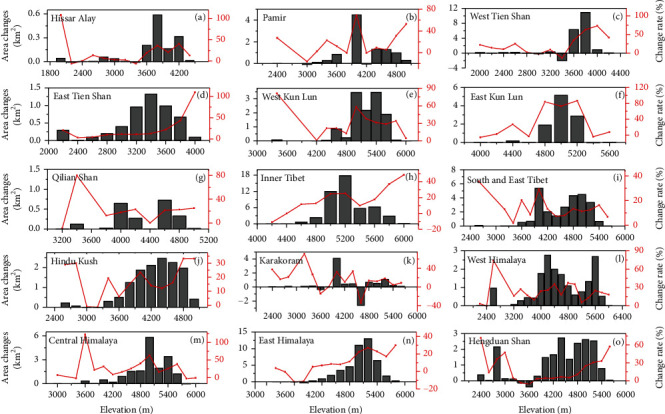
Changes in glacial lake area along the elevation (200 m bin sizes) for each subregion during 1990-2020.

Both area and number of glacial lakes have increased at different 200 m elevation bands in HMA (Figure S1). The greatest expansion occurred at 5000-5200 m a.s.l., with the increased area of about 45 km^2^ (~25%). The area change rate fluctuated at different elevation ranges with no obvious trend. The glacial lake area changes with elevation were significantly different among the different subregions (Figure 6). The change rates of glacial lakes in the West Tien Shan, East Tien Shan, Inner Tibet, and East Himalaya were found to be increasing with the increased elevation. However, the change rates varied markedly, and no significant trends of increase or decrease were observed in other subregions. In particular, glacial lakes in West Tien Shan and Karakoram exhibited large area decreases at elevations of about 3400 m a.s.l. (-1.99 km^2^) and 4600 m a.s.l. (-2.60 km^2^), respectively.

### 2.4. Effects of Climate and Glacier Thickness Changes on the Glacial Lake Changes

The heterogeneous changes in the glacial lake area in the whole HMA is closely related to complex climate changes and glacier activities. From 1990 to 2020, the temperature of all mountain ranges in HMA showed an increasing trend, with the highest warming rate of 0.6°C/10a (*P* value < 0.05) occurring in Inner Tibet and West Tien Shan (Figure S2(a)).

Rapid increase in the glacial lake area in HMA appears to be directly driven by multiple water sources, including net precipitation falling into the lakes, surface runoff from precipitation, glacier melting, and permafrost degradation. Almost the entire Himalaya Mountain ranges, as well as parts of the West Tien Shan, Hindu-Kush, and Hengduan Shan, are getting drier, while Inner Tibet is getting wetter (Figure S2(b)). Many studies have reported that ~70% of the increase in lake water in the endorheic basin (e.g., Inner Tibet) is due to the increased precipitation [50–52]. In the Himalayan region, precipitation decreased by up to 16 mm/a (*P* value < 0.05), so the precipitation in these regions did not account for the main contribution of glacial lake expansion.

At the same time, the mountainous glacier activities caused by the climate changes are intensifying, such as the rapid retreat of glacier ablation area, glacier surface thinning, and glacier surge [4, 53, 54]. Glacier changes affect the development and expansion of glacial lakes from several aspects. Glacier surface thinning provides source of water for the glacial lake area expansion, while the rapid retreat of the glacier terminus provides sufficient space for the development of glacial lakes. In this paper, to illustrate the effects of regional glacier surface elevation changes on the glacial lake changes, West Tien Shan, West Kun Lun, and East Himalaya were selected as the typical mountain regions due to the observed great total mass loss across the Tien Shan and Himalaya and significant positive mass change in the West Kun Lun [55–57]. As each subregion is very huge, it is difficult to show the glacier surface elevation changes at glacier-specific scale clearly. Therefore, partial regions in the three mountains with the most representative glacier changes and densely distributed glacial lakes surrounded are taken as examples (Figure S3). From 2000 to 2018, the glacier surface elevations in West Tien Shan and East Himalaya showed significant thinning, with the mean negative elevation changes of −0.24 ± 0.07 m/a and −0.39 ± 0.15 m/a, respectively, and the mean thinning rate of glaciers in East Himalaya was faster. During this period, the glacier surface elevation of West Kun Lun showed a thickening trend of 0.23 ± 0.10 m/a due to the extensive glacier surge [56]. Overall, it can be inferred that the rapid expansion of glacial lakes in HMA since 1990 is mainly driven by climate warming and glacier thinning, and the local evolution patterns of glacial lakes are related to the regional differences of climate variability and glacier ablation. For some regions with abnormal glacier thickness changes such as West Kun Lun, increased precipitation dominates the spatial distribution and variation of glacial lakes.

### 2.5. Evaluation of Extracted Glacial Lake Extent

**Figure 7:**
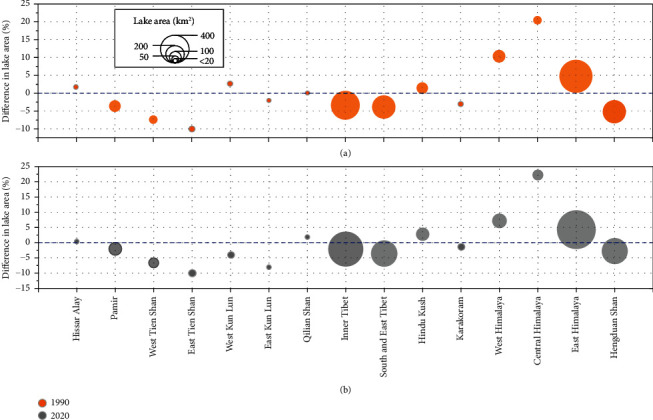
Differences in the extracted glacial lake areas in (a) 1990 and (b) 2020 with lake inventory from Wang et al. (2020). Bubble size denotes the total glacial lake area in each subregion.

The extracted glacial lake areas in 1990 and 2020 were compared with the newly released glacial lake inventory in 1990 and 2018, which was produced by Wang et al. (2020) over a much larger region, including Altai and Sayan. To make the comparison in the same spatial extent and minimum mapping unit, we excluded the Altai and Sayan region and lakes smaller than 0.0081 km^2^ from the inventory. The differences between the lake areas extracted in 1990 and 2020 and those derived from the inventory are illustrated in Figure 7.

**Figure 8:**
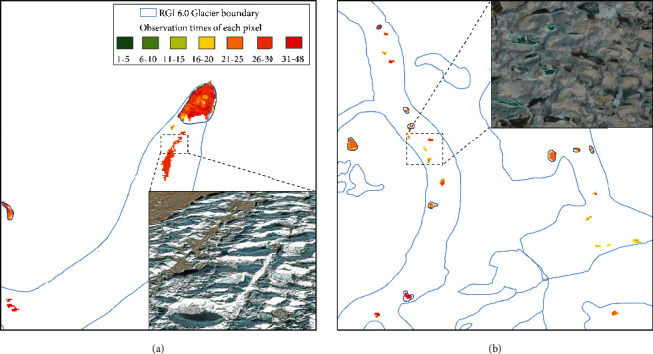
Examples showing the overestimated areas in Central Himalaya in 2020. (a) Snow and ice melt region on the glacier terminus; (b) supraglacial ponds formed on the debris-covered ablation zones. Dark blue polygons are the outlines from the glacial lake inventory. The two high-resolution Google Earth images are obtained on June 03, 2019, with supraglacial lakes shown in cyan outlines.

Our mapped results show general agreement with the inventory data for most of the subregions having varying total lake areas. The most overestimated lake area is located in Central Himalaya, which is shown as an example in Figure 8 for in-depth exploration. There are large glacial lake regions extracted by our proposed method which were not digitized as glacial lakes in the inventory. These regions are mostly distributed on the debris-covered ablation zones of glaciers below 3500 m. Our mapped glacial lake areas have a high degree of confidence, and the valid observation times of each pixel are usually higher than 10, meaning that they are not ephemeral water bodies and may exist throughout several warm seasons. In our opinion, this overestimation is acceptable due to the different definitions for glacial lake mapping and producer's operation. The substantial melting of glacier ice and snow was detected by our method (Figure 8(a)). Although they appeared as very shallow water with no clear water boundaries, they originated from glaciers and may be converted to supraglacial or proglacial lakes in the short term [5, 58]. Moreover, most of the water ponds lie on the low-altitude glacier tongue in our extracted results (Figure 8(b)), but in the glacial lake inventory in HMA from Wang et al. (2020), the highly dynamic seasonal supraglacial lakes that have relatively small sizes, complex shapes, and large quantities (even hundreds of supraglacial ponds on a glacier terminus) that are not easily digitized were partially excluded. They are the main cause for the large disagreements between the extracted glacial lake extent using our automated method and glacial lake inventory from Wang et al. (2020).

Additional comparisons in the total number, total area, and maximum/minimum area of glacial lakes with Wang et al. (2020) and Chen et al. (2021) are listed in Table S1. The statistics derived from our mapped glacial lakes are close to that of the inventory from Wang et al. (2020) for the epochs ~1990 and~2020. We found that thousands of glacial lakes with a total area of about 300 km^2^ were not detected by Chen et al. (2021). The main reasons for the missed glacial lakes in the inventory from Chen et al. (2021) are because of the interference of some bad observations (clouds or snow), drying up of small glacial lakes, or outburst in a single year [11].

### 2.6. Comparison of Lake Area Changes with Previous Studies

The studies about observations of glacial lake area changes in the whole HMA region are very limited and only focus on the two time periods [9, 10]. To fully validate our results in other periods, in this section, the detailed comparisons were performed using the reported data of glacial lake area changes for parts of HMA and time intervals. The wide area increases were documented in mountains such as Pamir, South and East Tibet, and Hengduan Shan during 1990–2010 [59–61]. In our extracted results, glacial lake areas in Pamir, South and East Tibet, and Hengduan Shan increased 0.58 ± 0.15%/a, 0.44 ± 0.11%/a, and 0.47 ± 0.19%/a from 1990 to 2010, respectively. The large-scale retreat and melting of glaciers is thought to be the major cause for the rapid expansion of lake area in these regions [13, 56]. In Inner Tibet, glacial lakes were reported to expand 0.86%/a during 1990–2000 [31]; in our results, glacial lakes increased 0.69 ± 0.22%/a during this period. For the whole Himalayan mountain range, we conducted a comparison between our results and the results from automatic extraction methods and human inspection during 1990–2015 [33]. In Nie et al.'s work, although each of the West Himalaya, Central Himalaya, and East Himalaya has been further divided into the northern side and southern side regions along the ridge line, respectively, it can be clearly shown that Central Himalaya expanded fastest, followed by the East Himalaya and West Himalaya over the past nearly three decades. This is similar to the results obtained by our method. Additional comparisons for different subregions and time intervals are listed in Table 1.

Although the area coverages are different, our results are consistent with previous results in most subregions and periods (Table 1). The time of the data in the collected references is usually close to the periods covered by our results. There are three possible reasons for the severe deviations in some subregions. In the Hindu Kush and Karakoram, the observed opposite trends of glacial lake changes from our results, and the reference data are possibly caused by the very small study area in the reference and the wide existence of surge-type glaciers in this region. Glacial lakes in East Kun Lun are small and very sparsely distributed; the different observation periods and lakes smaller than 0.01 km^2^ ignored by Zheng et al. (2021) may cause the differences in the results. In East Tien Shan, the large disagreement may stem from the snowy and rainy weather in areas above 3000 m, where ancient glacial deposits have also accumulated, seriously hindering the identification of glacial lakes. In the next section, we will discuss in more details about the influences of nonglacial lake areas on the mapping procedure.

## 3. Discussion

### 3.1. Influence of Snow and Ice

Most of the previous studies adopted visual interpretation and digitalization to delineate glacial lakes. Although snow/ice meltwater can be effectively excluded, lakes that are completely covered by snow or ice are still difficult to recognize from individual satellite image [42]. In this paper, we used a three-step strategy to identify frozen or snow-covered glacial lakes. First, Landsat images in the warm seasons are used. Second, glacial lakes might be covered with snow or ice many times, while over an ~five-year period, glacial lakes can be identified based on the stable status of water bodies (NDWI > 0.1). Third, the observation times for most of the regions in HMA are greater than 10, which improves the reliability of multitemporal glacial lake mapping results.

The proposed method shows good performance for the large-scale glacial lake extraction even in the monsoon-affected areas with heavy cloud cover; e.g., Hengduan Shan and South and East Tibet, their differences with the glacial lake inventory are less than 5% (Figure 7). In Central Himalaya, it was also found that there is a wide coverage of supraglacial ponds or snow/ice meltwater that were not delineated as glacial lakes in the inventory (Figure 8). Therefore, to reduce the bias for the estimation of glacial lake area changes, we take the intersection areas of our extracted glacial lakes with glacier boundaries as the uncertainty area. At present, however, we think it is difficult to resolve the discrepancy between our results and manual inventory since variation in the snow or ice melt contributions is hardly ignored during the automatic extraction over such a large area.

### 3.2. Influence of Topographic Shadow

The influence of topographic shadow in mountainous area is still a common problem for the delineation of glacial lakes or glaciers. Topographic parameters like shaded relief and slope are usually employed to distinguish between terrain shadows and lakes [11, 50]. However, the influence of terrain shadow cannot be completely eliminated due to the insufficiently detailed topographic features and time lag of DEM data.

**Figure 9:**
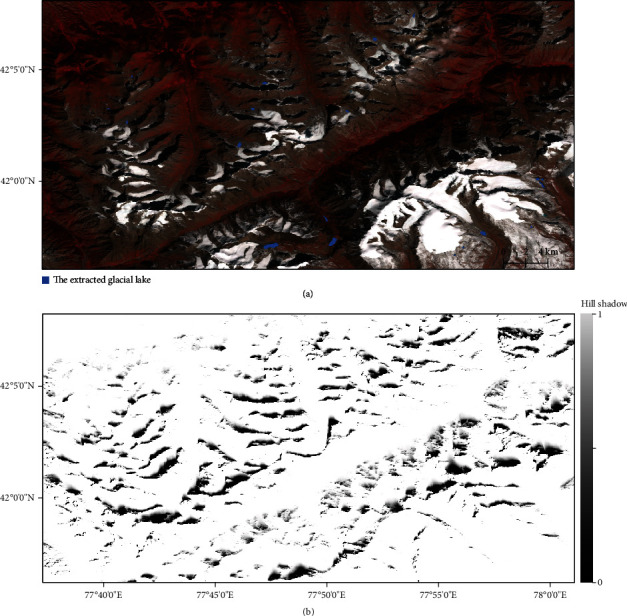
Hill shadow results in West Tien Shan in 2020. (a) Landsat 8 OLI image is acquired on September 26, 2020, with extracted glacial lakes shown in blue; (b) mean hill shadow map calculated using the image collection (images in warm seasons during 2018–2021) in GEE. The lower the value, the higher the probability of being a hill shadow.

The shadow area in the remote sensing image is mainly determined by the sun elevation and topography when the image is collected. In HMA, the areas seriously affected by the terrain shadows are the mountains with east–west ridges or near east–west ridges, such as the Tien Shan Mountains (Figure 9(a)), for which glaciers typically appear along the northern slope [40]. To greatly reduce topographic shadow, imagery collected from September to October is preferentially used because the solar elevation angles are relatively high. In our case, we fully consider the topographic relief, solar azimuth, and zenith synchronous with the image acquisition and use the mean value of the hill shadow for each time-series pixel set to alleviate shadowing effect (Figure 9(b)). It should be noted that the areas of the shadowed surface are different over different regions and even in the same region are different at different times, but generally, the remaining shadowed surface after the processing using the slope and mean hill shadow occupies a very small proportion in the final extracted lakes and will seldom affect the estimation of glacial lake area changes after subtraction of different periods.

### 3.3. Limitations and Advantages of the Proposed Method

Multitemporal image composite was necessary for the low-quality images affected by thick clouds, frozen and snow-covered lakes, and topographic shadows. Glacial lake extent extracted using our automated method fits the real boundaries of the glacial lakes very well (Figure S4), while manually delineated glacial lake outlines are largely influenced by people's subjective experience and manual operation, resulting in inaccurate extraction of lake details near the glacier terminus and overestimation or complete omission of some small glacial lakes. Moreover, manual digitization shows poor performance for the delineation of glacial lakes with complex curved shapes.

Apart from manual delineation, many automatic methods were proposed for the glacial lake extraction. Thresholding segmentation method is simple but effective for the small homogeneous area [9, 19, 33, 40], while for a large region with diverse environment, the classification accuracy will be greatly reduced. To improve the details of lake edge, level-set-based active-contour approaches fully consider the regional heterogeneity and can be applicable to different features [15, 22, 65, 66]. However, the calculation is complicated, and many attempts are needed to select optimized parameters. Object-oriented classification methods full exploit the lake spectral information, morphological characteristics, and contextual information [21, 23]. The main limitation lies in the manual establishment of classification rules and segmentation scales. The improved deep learning methods have high automation for detecting glacial lakes without assistance of additional auxiliary data [24–26, 67], while enough and representative training samples are required.

In comparison with these automatic glacial lake mapping methods, our method has the following advantages: (1) The method was developed based on the GEE cloud computing platform which offered massive images for the high-efficiency pixel-level composite. (2) It is unnecessary to select available data from a large number of images and then merge them; all images can be fully utilized to obtain a pixel-level composited glacial lake map. (3) The impacts of ice, snow, cloud, and hill shadow were comprehensively considered and greatly removed; the evaluation indicators were established to reduce the impact from glacial meltwater on the mapping results. (4) Our method shows high accuracy for the extraction of the lake details and can capture areas of tiny supraglacial and unconnected glacial lakes that are easily overlooked.

However, there are also some limitations in the proposed method. The main problem with compositing images over ~5 years is that in regions where glacial lakes change extremely rapidly, the edges of glacial lakes will look “blurred.” For the few highly active glacial lakes, e.g., Lake Merzbacher in Tien Shan, which has suddenly breached nearly every year due to intense glacier melting or huge ice falling into the lake [8], our method is not able to capture rapid changes and ensuing GLOF events over the entire growing period of such glacial lakes. That is the main limitation of this methodology in detecting glacial lake changes, which only considers most of glacial lakes that evolve stably or slowly.

Another problem with our proposed method is that we consider all the lakes within this 10 km buffer zone to be glacial lakes, which is controversial and may cause deviations since nonglacier-fed lakes were regarded as nonglacial lakes in some studies [31, 32]. Examples of the deviations caused by the nonglacial lake pixels in typical regions are shown in Figure S5. It can be seen that nonglacial lakes bring great uncertainty to the total number and area of glacial lakes in the East Himalaya. The number of nonglacial lakes accounts for nearly half of the total number; the area accounts for a slightly lower proportion, about 18%. The errors caused by the nonglacial lake area in West Tien Shan and West Kun Lun decreased, occupying 8% and 2% of the total lake area, respectively. More deviations of number were observed for the West Tien Shan, with nonglacial lakes accounting for about 18% of the total number. Generally, the errors caused by the nonglacial lake pixels are different in different mountains, and the resulting errors of area are smaller than that of the number.

In the mapping results, glacial lakes directly in contact with glaciers or lying on the glacier surface were identified; however, some glacial meltwater at the glacier terminus was also extracted with high confidence (the observation times were usually >10), excluding the possibility of transient meltwater (Figure 8). This glacial meltwater can exist for several warm seasons and will continue to accumulate or flow downward [68]. Traditional methods using human vision have difficulty in distinguishing glaciers from glacial meltwater, but glacial meltwater can be quickly differentiated using our proposed method. Although this continuous melting of snow and ice has not yet evolved into a stable water body and has not been taken as glacial lake in the manually delineated inventory, in our opinion, it is more sensitive to the climatic warming than glacier itself due to the thermal effect and offers new perspectives for understanding the glacier mass loss and glacial lake formation.

## 4. Conclusion

No systematic studies have been made to investigate the current distribution and long-term heterogeneous changes of glacial lakes in the whole HMA. Glacial lake mapping from satellite images is of crucial importance for glacial lake dynamics monitoring. In previous studies, the heavy clouds, snow or ice cover, and terrain shadows were the major obstacles for image selection from a large number of images and accurate lake mapping. This study is aimed at solving these problems by developing a new automated glacial lake extraction method. First, due to the spectral complexity and multiple influencing objects in alpine environments, the multitemporal mean NDWI composite was proposed for the stable glacial lake extraction. Then, different cloud scores and NDSI settings were tested in this study, and shadowed pixels were greatly reduced using the mean hill shadow > 0.9. The uncertainties from supraglacial lakes and glacial meltwater were estimated using the intersection area between the extracted results and glacier inventory boundaries. The strength of this method is that we composite the glacial lake image at the pixel level by making full use of all the available images, rather than just image mosaicking.

Glacial lake area changes present successive and consistent increases in the past 30 years. The estimated glacial lake area increase was 0.58 ± 0.21%/a (8.57 ± 3.16 km^2^/a) in HMA from 1990 to 2020, showing strong spatiotemporal heterogeneity of changes. The fastest expanding area of glacial lake was East Kun Lun, and its growth rate was 2.01 ± 0.54%/a. Pamir and Hengduan Shan have the slowest increases, which were 0.33 ± 0.08%/a and 0.39 ± 0.01%/a, respectively. Even in the glacier accumulated regions—West Kun Lun—the glacial lake area increased to some extent due to the dominant effects from the increased precipitation. The greatest expansion occurred at 5000-5200 m a.s.l., with the increased area of about 45 km^2^ (~25%). The warming temperature and thinning glaciers are considered to be the main drivers of widespread glacial lake expansion in HMA region. The long-term glacial lake dataset, proposed automated glacial lake mapping method, and findings in this study have important implications to improve the understanding of glacial lakes and glacier hydrological changes, as well as for disaster risk assessment in mountainous glaciated regions.

## 5. Materials and Methods

### 5.1. Datasets

Approximately 30,000 Landsat Thematic Mapper (TM) and Operational Land Imager (OLI) scenes were accessed from GEE for the lake mapping in the periods of 1990, 2000, 2010, and 2020. The ten-year interval between these four periods is chosen to fully explore the changes in glacial lakes during the past decades. Because the high-quality images in a single year are quite limited in HMA, to obtain abundant data, all available images were collected within ±2~3 years of the four periods (Table 2). Landsat 8 shows better performance for the lake mapping compared with its previous sensors due to the enhanced image acquisition technology and improved sensor [69]. The archived Landsat 5 images are fewer than those of Landsat 8, and thus, the selected time span for 1990 and 2000 is relatively long. Landsat Tier 1 calibrated top-of-atmosphere (TOA) reflectance images on GEE were employed.

Other auxiliary dataset are as follows: (i) Randolph Glacier Inventory (RGI v6.0) [30] to delimit the distribution of glacial lakes within its 10 km buffer. An updated glacier inventory—Glacier Area Mapping for Discharge from the Asian Mountains (GAMDAM) [70]—has also been included to supplement some missing glaciers in RGI v6.0. (ii) HMA glacial lake inventory for comparison and cross-validation of our results [10]. (iii) Version 3.0 Shuttle Radar Topography Mission (SRTM) DEM with approximately 30 m resolution to reduce the effect of terrain shadows on glacial lake mapping and combined with TanDEM-X data acquired in 2018, to calculate the glacier thickness changes (TanDEM-X minus SRTM DEM). (iv) Monthly gridded temperature dataset derived from Climate Research Unit (CRU) data (v4.05, 0.5° spatial resolution) to estimate the temperature changes in HMA from 1990 to 2020. (v) Monthly land-surface precipitation data from Global Precipitation Climatology Centre (GPCC) with a spatial resolution of 0.25° to estimate the precipitation trend in HMA during 1990–2019.

### 5.2. Methodology

#### 5.2.1. The Processing Chain

**Figure 10:**
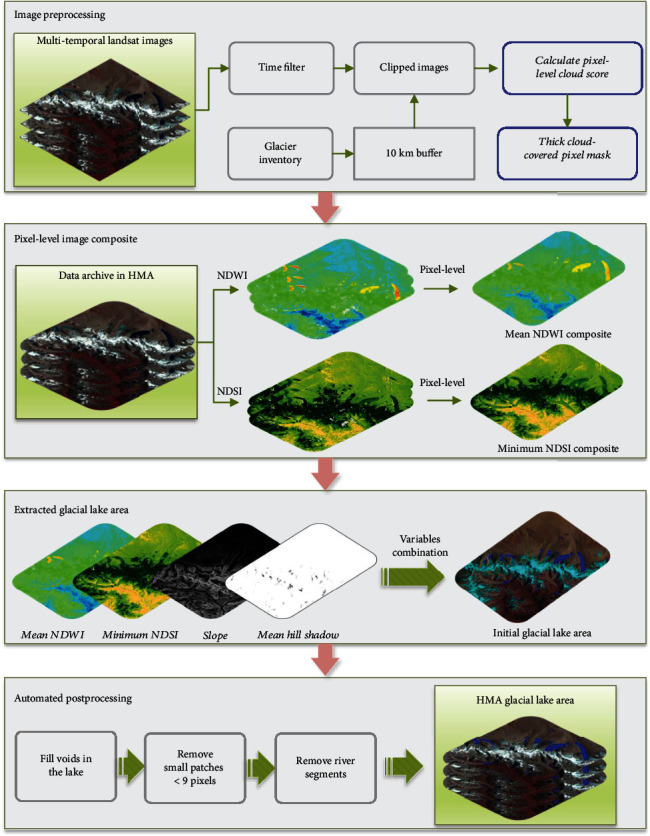
Flowchart of the processing chain for automated glacial lake detection.

To automatically detect glacial lakes from Landsat time series of images while overcoming the limitations of clouds, terrain shadows, and seasonal snow or ice cover, our proposed method composites a glacial lake map on pixel scale, which mainly consists of four steps (Figure 10): (1) image preprocessing to filter out the frozen season images, nonglacial lakes, and thick cloud-covered pixels; (2) multitemporal mean NDWI composite and minimum NDSI composite; (3) glacial lake area extraction; and (4) automated postprocessing step.

*(1) Image Preprocessing*. To minimize the influences from seasonal snow or ice cover, images were mostly selected in the warm season (June to November) for the four periods. During this time, the lakes also featured the most stable and maximum extent following glacier ablation. In monsoon-affected regions such as Southeastern Tibet and Central and Eastern Himalaya, late September to November is more suitable due to the abundant monsoon cloud cover in summer. We reduced the search range to <10 km of glacier terminus in the inventory and considered all the mapped lakes within this range as glacial lakes [10, 33]. We excluded areas far from each glacier, assuming that plateau lakes in these regions have weak interactions with glaciers.

**Figure 11:**
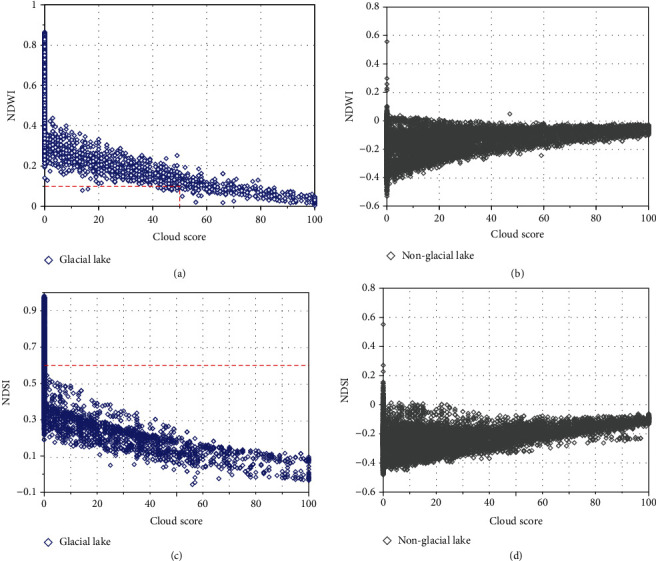
Relationship between cloud score, NDWI, and NDSI value for (a, c) 11,715 glacial lake pixels within the lake inventory boundaries and (b, d) 12,819 nonglacial lake pixels, all of which are selected visually from Landsat images in Karakoram and Central Himalaya with varying degrees of cloud cover.

Time series of clipped Landsat images in HMA make up pixel sets at each pixel location. Then, the cloud score (in the range [0, 100]) of each pixel is calculated using the Landsat simpleCloudScore function provided by GEE based on a combination of brightness, temperature, and NDSI. For the cloudy areas, the absolutely clear-sky pixels (cloud score equals zero) are too limited to composite a glacial lake map. We found that water covered by thin clouds can be separated from the land and still be identified as water pixels. Here, we used 11,715 pixels of lakes located within the boundaries of glacial lake inventories from Wang et al. (2020), pixels that may be cloudy or clear; it was observed that NDWI decease with the increase of cloud score (Figure 11(a)). For glacial lake pixels with cloud scores less than 50, their NDWI is higher than 0.1, the threshold within the range of 0.05 to 0.15 for glacial lake mapping [19, 22]. The results for nonglacial lake areas show no obvious trend (Figure 11(b)), but the NDWI is mostly lower than 0, indicating that nonglacial lake pixels are less likely to be recognized as glacial lake pixels due to the cloud score settings. Some glaciers connected to glacial lakes can be misclassified as lakes using only NDWI; thus, NDSI was utilized for the further glacial lake identification. The NDSI of most lake pixels is lower than 0.4 (Figure 11(c)), a threshold that is generally used for the extraction of glacier coverage area [49, 71]. Almost all pixels are lower than 0.6, which may be more reasonable for the exclusion of glaciers while extracting lake water bodies that are sometimes partially covered with ice or snow. The NDSI of nonglacial lake regions is insensitive to the cloud score, similar to the results of NDWI (Figure 11(d)). Based on the above analysis, we used a threshold of 50 on the cloud score to mask thick cloudy pixels. A very low cloud score threshold will reduce the available pixel sets, and seasonal snow and ice melt will be misclassified as glacial lakes, resulting in overestimation of lake area. A very high threshold will include thick cloud pixels, which will be identified as land pixels no matter the clouds are over land or water [72], making the lake area underestimated.

*(2) Pixel-Level Image Composite*. This step is aimed at calculating the NDWI and NDSI values for each pixel of the images after the cloud mask and then composite new images with the mean NDWI value and minimum NDSI value for each pixel set. The mean NDWI value represents the stable status of lake pixels, and it can obtain complete interior pixels of lake water to the greatest extent. The minimum NDSI is helpful for the exclusion of snow or ice cover when the exposed glacier is undergoing ablation during the observation time.

*(3) Glacial Lake Area Extraction*. According to the above experiments and related studies [19, 22], glacial lakes in the Tibetan Plateau have NDWI values higher than 0.05 or 0.15 using Landsat images. Here, we set the threshold of NDWI to 0.1 to extract glacial lakes from the composited image. To avoid commission errors from the glacier and, at the same time, retain the lakes partly covered by seasonal snow or bare ice, 0.6 is used as the threshold on the minimum composited NDSI image. A slope threshold of <20° [9] is employed to alleviate disturbances from terrain shadows. Moreover, shadow of each pixel set is calculated using the hillShadow algorithm in GEE taking the input of the DEM, solar azimuth, and zenith of the image, with an output of 1 where pixels are illuminated and 0 where they are shadowed. In this study, pixels with mean hill shadows higher than 0.9 are considered illuminated regions given the transient appearance of shadows on the time scale. By synthesizing from the former conditions, binary images of glacial lakes in HMA are obtained.

*(4) Automated Postprocessing*. In the binary image, some of the holes exist within the lakes due to the heterogeneous spectral responses from variable suspended solids, water depth, and colored dissolved materials in the water. In a postprocessing step, we search the connected void pixels in the existing lake region and fill them and remove small patches < 9 pixels (0.0081 km^2^) that originate from noise from unstable tiny ponds and river segments [11]. Finally, the time series of glacial lake maps are generated. This method has a high degree of automation for the glacial lake extraction. Given the amount of work required to update the inventory and improve the work efficiency of large-scale glacial lake dynamic monitoring, while minimizing the influence of subjective judgment of operators, each glacial lake boundary was not visually checked and reedited with original Landsat images.

#### 5.2.2. Tests of Optimal NDWI Threshold

In HMA, glacier backgrounds dominate the scenes. Therefore, both NDWI and NDSI are applied sequentially: firstly, NDWI is applied to extract the water bodies from images; then, NDSI is used to eliminate the pixels misclassified with glaciers. Since NDWI is designed to separate water and nonwater types, it forces water types above 0 and nonwater types below 0; some thresholds above 0 (e.g., 0.05 or 0.15) are applied to extract glacial lakes from NDWI images. However, due to the varying brightness and contrast of the scene with space and time, these thresholds might not always achieve the best classification accuracy. In this section, multiple thresholds of NDWI were considered to determine the optimal threshold, which approximates to the minimum sum of commission and omission errors.

**Figure 12:**
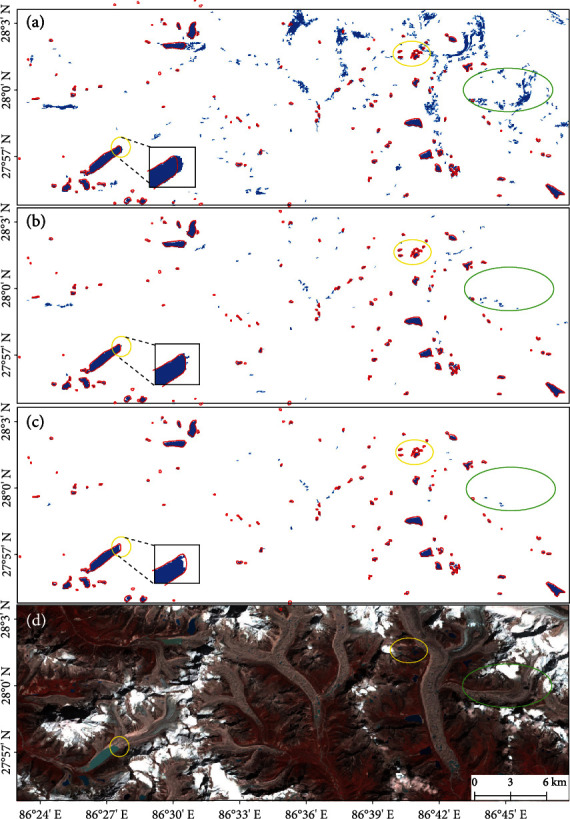
Glacial lake extraction images applying NDWI at (a) 0.05, (b) 0.1, and (c) 0.15 with NDSI at 0.6 in the East Himalaya in 2020. (d) is the corresponding false color composite of the Landsat 8 OLI image on September 05, 2020. The extracted glacial lakes are shown in blue, and the red outlines are the 2018 glacial lake inventory delineated by Wang et al. (2020). The areas within the yellow ellipses are considered as glacial lakes in the inventory, but using different thresholds of NDWI, parts of the area are not identified as glacial lakes. The areas within the green ellipses are areas outside the inventory, while some may actually be omitted by the inventory, and some might be identified as glacial lakes after applying different thresholds.

A comparison of the glacial lake extraction results using different thresholds of NDWI is shown in Figure 12 as an example. The glacial lake outlines in 2018 [10] generated by satellite images from 2016 to 2020 were taken as a reference. Obviously, the threshold of NDWI at 0.05 (Figure 12(a)) overestimated the lake area; although most areas within the inventory boundaries are identified as glacial lakes, in the regions outside the inventory boundaries, large portions of glacier terminus are also mistakenly recognized. If the threshold of NDWI is set to 0.15 (Figure 12(c)), the influence of glaciers is greatly reduced, but many small glacial lakes are not detected, and there are missing pixels on the lake edge. This implies that 0.1 (Figure 12(b)) could be reasonable to classify glacial lakes under various environmental conditions, with a tradeoff between commission and omission errors. The choice of the multitemporal pixel-level image composite scheme also intends to stabilize this threshold.

#### 5.2.3. Tests of Cloud Score Thresholds

**Figure 13:**
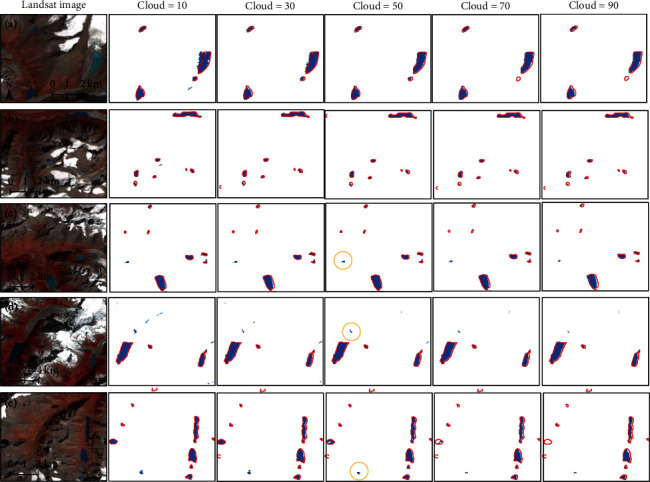
Experiments for different thresholds of cloud score (cloud score is set to 10, 30, 50, 70, and 90) on the composited image of 2020 at five test sites in the South and East Tibet. Glacial lakes extracted by our method are shown in blue color, and red outlines show the glacial lake inventory in 2018 (Wang et al., 2020). The regions in yellow ellipses are the lakes not identified by the glacial lake inventory. The central longitude and latitude of the five sites are (a) 93.5182°E, 30.4753°N; (b) 93.4925°E, 30.4092°N; (c) 93.4610°E, 30.2945°N; (d) 94.1435°E, 30.1417°N; and (e) 93.2521°E, 30.1124°N.

The use of different thresholds of the cloud score directly controls the number of available pixels in each pixel location; thus, it is important for the final glacial lake extraction. An example of the influence of different cloud score thresholds on glacial lake extraction is presented in Figure 13. In HMA, South and East Tibet is one region most vulnerable to cloud cover, so the composited image in 2020 in the South and East Tibet region is selected as an example. Generally, most glacial lakes can be identified using this pixel-level composite strategy regardless of the cloud score. It can even automatically extract previously undiscovered glacial lakes (shown in the yellow ellipses). When the threshold of cloud score is below 50, clouds have little impact on glacial lake extraction. Most pixels in the inventory boundaries are delineated, but many pixels of snow or ice are misclassified as glacial lakes. When the cloud score is higher than 50, thick clouds block all information for land (including snow and ice) and water separation, and glacial lake extraction is prone to be underestimated together with the absence of small glacial lakes. The cloud score should therefore be set to 50 to ensure a satisfactory amount of the pixel sets used and high extraction accuracy.

#### 5.2.4. Uncertainties of Snow or Ice Melt

**Figure 14:**
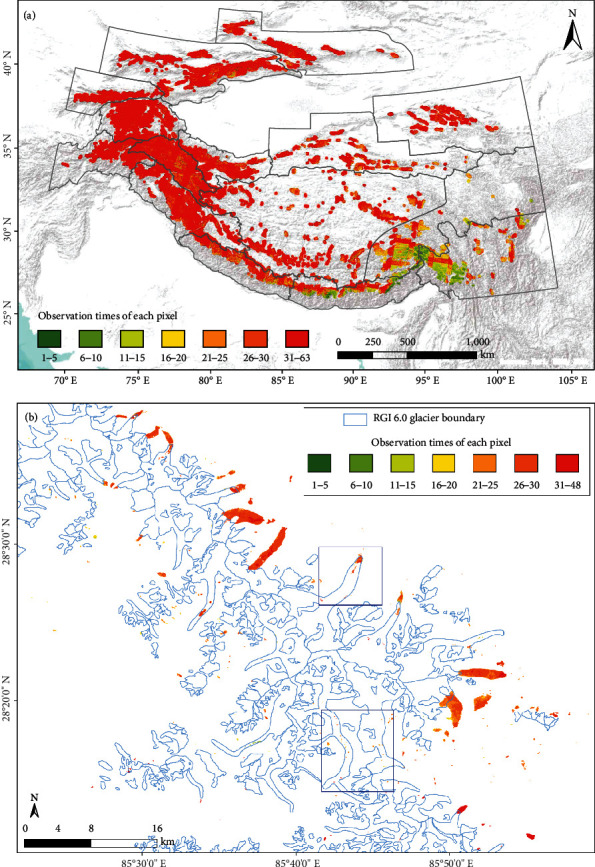
(a) Map of observation times per pixel after cloud filtering for the glacier-buffered regions in HMA. Black polygons represent the extent of subregions. (b) Lake-specific mapping times in the Central Himalaya in 2020. Two blue boxes mark the location of maps enlarged in Figure 8.

Glacial lake area could be overestimated due to the melting of ice or snow on the glaciers if the data were found to be insufficient (Figure 13). Here, the mean NDWI value of pixel set is taken as corresponding value of composite images, meaning that if seasonal meltwater has been short-lived, those pixels would not be recognized as glacial lakes. We also calculated the observation times of each pixel used in the production of the multitemporal mean NDWI composite and minimum NDSI composite, which records how many valid pixels in each pixel location can be used to determine whether a pixel is a glacial lake during the observation period (Figure 14(a)). The observation times are high on most occasions because the data were collected during ~5 warm seasons (June to November) with a revisit cycle of 16 days for each mapping period (Table 2). However, in areas with frequent cloud cover, such as Himalaya and South and East Tibet regions, the observation times may be relatively low after the cloudy pixels are masked. Using the combination of NDWI and NDSI, most of the pixels of glaciers distributed downstream or around glacial lakes can be excluded, but there are still a few misclassified glacier pixels with sustained high water content, usually known as glacier and snow runoff. Figure 14(b) is taken as an example to show the observation times of each pixel in the mapped glacial lake areas in Central Himalaya. More observation times mean that glacial lakes can be more accurately extracted, but this also inevitably mixes with some high glacial meltwater pixels. To reduce noise and ensure the quality of glacial lake changes, one possible solution is to directly mask off glaciers using a glacier outline inventory. However, such work might also erase much of the information about supraglacial lakes that lie on the surface of glaciers. In this study, we take the intersection area of the glacier boundary and extracted glacial lake areas as qualitative measurement of area uncertainties, by fully considering the impact of glacial meltwater and the uncertainty caused by the high variability of the supraglacial lakes.

## Figures and Tables

**Table 1:** Comparison of glacial lake area changes derived by our proposed method and previous studies. Statistics at different times in the columns of number and area are separated by slash. “-” indicates that data are not recorded.

Subregion	This study				References					
	Time interval	Rate (%/a)	Number	Area (km^2^)	Time interval	Rate (%/a)	Number	Area (km^2^)	Study area	Source
Hissar Alay	1990–2020	0.45 ± 0.12	112/137	9.87 ± 1.97/11.20 ± 2.14	1990–2015	0.36	176/224	14.5 ± 0.2/15.8 ± 0.1	The whole region	[9]
Pamir	1990–2010	0.58 ± 0.15	597/665	96.19 ± 18.44/107.52 ± 23.65	1990–2013	0.45	238/188	78.01 ± 2.74/86.07 ± 2.67	West Tarim basin in the Pamir	[59]
West Tien Shan	1990–2010	0.62 ± 0.24	954/1124	69.90 ± 18.52/78.53 ± 21.65	1990–2010	0.58	990/1146	60.64 ± 8.68/67.73 ± 9.84	The whole region	[45]
					1972–2007	0.98	66/132	2.56 ± 0.14/3.44 ± 0.18	Two Landsat scenes	[62]
East Tien Shan	1990–2010	0.42 ± 0.08	586/714	31.11 ± 7.55/33.76 ± 6.52	1990–2010	1.51	371/521	22.08 ± 3.19/28.79 ± 4.39	The whole region	[45]
West Kun Lun	1990–2010	0.59 ± 0.30	131/143	33.17 ± 5.19/37.14 ± 6.29	1990–2013	1.21	217/179	6.98 ± 1.10/8.92 ± 1.19	Tarim basin in the West Kun Lun	[59]
East Kun Lun	1990–2020	2.01 ± 0.54	309/391	13.90 ± 3.38/22.30 ± 4.36	1990–2015	0.24	182/181	9.8 ± 0.2/10.4 ± 0.1	The whole region	[9]
Qilian Shan	1990–2020	0.80 ± 0.05	159/207	11.17 ± 2.65/13.86 ± 2.77	1990–2015	1.29	112/130	6.2 ± 0.1/8.2 ± 0.1	The whole region	[9]
Inner Tibet	1990–2000	0.69 ± 0.22	3389/3552	239.05 ± 40.04/255.65 ± 47.16	1990–2000	0.86	275/300	28.9 ± 4.6/31.4 ± 5.0	Inner Plateau basin	[31]
South and East Tibet	1990–2010	0.44 ± 0.11	2762/2914	193.68 ± 35.96/210.67 ± 37.95	1988–2013	0.38	1278/1396	85.24/93.31	One Landsat scene	[60]
Hindu Kush	2000–2010	0.12 ± 0.04	1842/1853	97.68 ± 19.81/98.91 ± 17.22	2001–2013	-0.11	-/722	51.4/50.7	Hindu Kush in Pakistan	[63]
Karakoram	1990–2000	0.36 ± 0.08	534/596	36.06 ± 7.33/37.38 ± 7.48	1990–2000	-3.6	—	—	One Landsat scene	[14]
	2000–2010	0.67 ± 0.17	596/629	37.38 ± 7.48/39.90 ± 9.15	2000–2009	1.0	-/422	-/3.7	One Landsat scene	[14]
					2001–2013	1.64	-/1325	46.2/55.3	Karakoram in Pakistan	[63]
	1990–2010	0.53 ± 0.16	534/629	36.06 ± 7.33/39.90 ± 9.15	1990–2013	0.61	162/141	14.74 ± 1.13/16.81 ± 1.09	Tarim basin in the Karakoram	[59]
West Himalaya	1990–2020	0.51 ± 0.31	1525/1895	105.08 ± 20.35/121.30 ± 24.67	1990–2015	0.22	-/979	-/50.0	North of ridge line	[33]
					1990–2015	0.20	-/476	-/38.8	South of ridge line	
Central Himalaya	1990–2020	0.91 ± 0.11	1055/1304	70.68 ± 14.14/89.92 ± 16.34	1990–2015	0.55	-/839	-/117.4	North of ridge line	[33]
					1990–2015	0.92	-/1104	-/86.3	South of ridge line	
					1988–2015	2.00	107/148	4.62/7.12	Gyirong River basin	[64]
East Himalaya	1990–2020	0.57 ± 0.22	3189/3611	274.21 ± 44.95/321.46 ± 54.29	1990–2015	0.31	-/502	-/66.0	North of ridge line	[33]
					1990–2015	0.44	-/1050	-/96.8	South of ridge line	
Hengduan Shan	1990–2010	0.47 ± 0.19	2279/2564	193.05 ± 35.75/211.43 ± 38.44	1990–2014	0.12 ± 0.03	2872/3235	248.9 ± 29.9/255.8 ± 31.6	The whole region	[61]

**Table 2:** Information of the datasets used in this study.

Dataset	Temporal coverage	Spatial resolution	Data type	Source
Landsat imagery	1990 (1988–1993)	30 m	Landsat 5/TM, tier 1 TOA	United States Geological Survey (USGS) https://earthexplorer.usgs.gov
	2000 (1998–2003)	30 m	Landsat 5/TM, tier 1 TOA	
	2010 (2009–2012)	30 m	Landsat 5/TM, tier 1 TOA	
	2020 (2018–2021)	30 m	Landsat 8/OLI, tier 1 TOA	
SRTM DEM	2000	30 m	Single-raster band	USGS https://http://earthexplorer.usgs.gov
TanDEM-X	2018	1.7-3.5 m	Single-raster band	Apply from German Aerospace Center (DLR) https://tandemx-science.dlr.de/
RGI v6.0 Region 13–15	1999–2010	—	Multipolygon shapefile	Global Land Ice Measurements from Space (GLIMS) https://www.glims.org/RGI/rgi60_dl.html
GAMDAM glacier inventory	1990–2010	—	Multipolygon shapefile	See Sakai (2019)
CRU v4.05	1990–2020	0.5°	Gridded dataset	https://crudata.uea.ac.uk/cru/data/hrg/
GPCC, full data monthly version 2020	1990–2019	0.25°	Gridded dataset	https://opendata.dwd.de/climate_environment/GPCC/html/download_gate.html

## Data Availability

The data used to support the findings of this study are included within the article and can be requested from the authors.

## Supplementary Materials

Supplementary Materials Figure S1: changes in the area and number of glacial lakes for each 200 m elevation band in HMA region from 1990 to 2020: (a) area changes; (b) number changes. Figure S2: spatial pattern of temperature and precipitation changes in HMA: (a) trend in temperature change from 1990 to 2020 produced by CRU data (v4.05, 0.5° spatial resolution); (b) annual mean precipitation changes for the period 1990–2019 obtained from GPCC data (full data monthly version 2020, 0.25° spatial resolution). The symbol “+” indicates the linear fit of temperature/precipitation versus time is at the 90% confidence interval. Figure S3: glacier surface elevation changes in three typical glaciated areas of HMA during 2000–2018: (a) West Tien Shan; (b) West Kun Lun; (c) East Himalaya. The values in subplots represent the estimated mean glacier elevation changes in each region. Figure S4: example showing the mapped glacial lakes by our purposed method, Chen et al. (2021), and Wang et al. (2020) in the Central Himalaya in 2020. (a–d) represent four enlarged maps of glacial lakes. Background image is from false-color composited (bands: 5/4/3) Landsat-8 OLI image in October 2, 2020. Figure S5: examples showing the deviations caused by the nonglacial lake pixels: (a) geographic location of three typical regions; (b) number and area of all the mapped lakes and nonglacial lakes for typical regions in West Tien Shan, West Kun Lun, and East Himalaya. Table S1: comparisons between the two released HMA glacial lake inventories and glacial lakes mapped in this study.
