# Supplementary material for: Glacial Lake Area Changes in High Mountain Asia during 1990–2020 Using Satellite Remote Sensing
Source: Research (Wash D C). 2022 Oct 21;2022:9821275. doi: 10.34133/2022/9821275 (PMC9639448; doi:10.34133/2022/9821275)
Supplement: Supplementary Materials — Figure S1: changes in the area and number of glacial lakes for each 200 m elevation band in HMA region from 1990 to 2020: (a) area changes; (b) number changes. Figure S2: spatial pattern of temperature and precipitation changes in HMA: (a) trend in temperature change from 1990 to 2020 produced by CRU data (v4.05, 0.5° spatial resolution); (b) annual mean precipitation changes for the period 1990–2019 obtained from GPCC data (full data monthly version 2020, 0.25° spatial resolution). The symbol “+” indicates the linear fit of temperature/precipitation versus time is at the 90% confidence interval. Figure S3: glacier surface elevation changes in three typical glaciated areas of HMA during 2000–2018: (a) West Tien Shan; (b) West Kun Lun; (c) East Himalaya. The values in subplots represent the estimated mean glacier elevation changes in each region. Figure S4: example showing the mapped glacial lakes by our purposed method, Chen et al. (2021), and Wang et al. (2020) in the Central Himalaya in 2020. (a–d) represent four enlarged maps of glacial lakes. Background image is from false-color composited (bands: 5/4/3) Landsat-8 OLI image in October 2, 2020. Figure S5: examples showing the deviations caused by the nonglacial lake pixels: (a) geographic location of three typical regions; (b) number and area of all the mapped lakes and nonglacial lakes for typical regions in West Tien Shan, West Kun Lun, and East Himalaya. Table S1: comparisons between the two released HMA glacial lake inventories and glacial lakes mapped in this study. [file 9821275.f1.docx]

**Supplementary Materials**

Glacial Lake Area Changes in High Mountain Asia during 1990–2020 Using Satellite Remote Sensing

Meimei Zhang ^1,2^, Fang Chen ^1,2,3^*, Huadong Guo ^1,2,3^, Lu Yi ^4^, Jiangyuan Zeng ^5^, and Bin Li ^1,2^

^1^ International Research Center of Big Data for Sustainable Development Goals, Beijing 100094, China

^2^ Key Laboratory of Digital Earth Science, Aerospace Information Research Institute, Chinese Academy of Sciences, No. 9 Dengzhuang South Road, Beijing 100094, China

^3^ University of Chinese Academy of Sciences, Beijing 100049, China

^4^ Key Laboratory of Coastal Environment and Resources Research of Zhejiang Province, School of Engineering, Westlake University, Hangzhou 310024, China

^5^ State Key Laboratory of Remote Sensing Science, Aerospace Information Research Institute, Chinese Academy of Sciences, Beijing 100094, China

*Correspondence should be addressed to Fang Chen; [chenfang_group@radi.ac.cn](mailto:chenfang_group@radi.ac.cn)


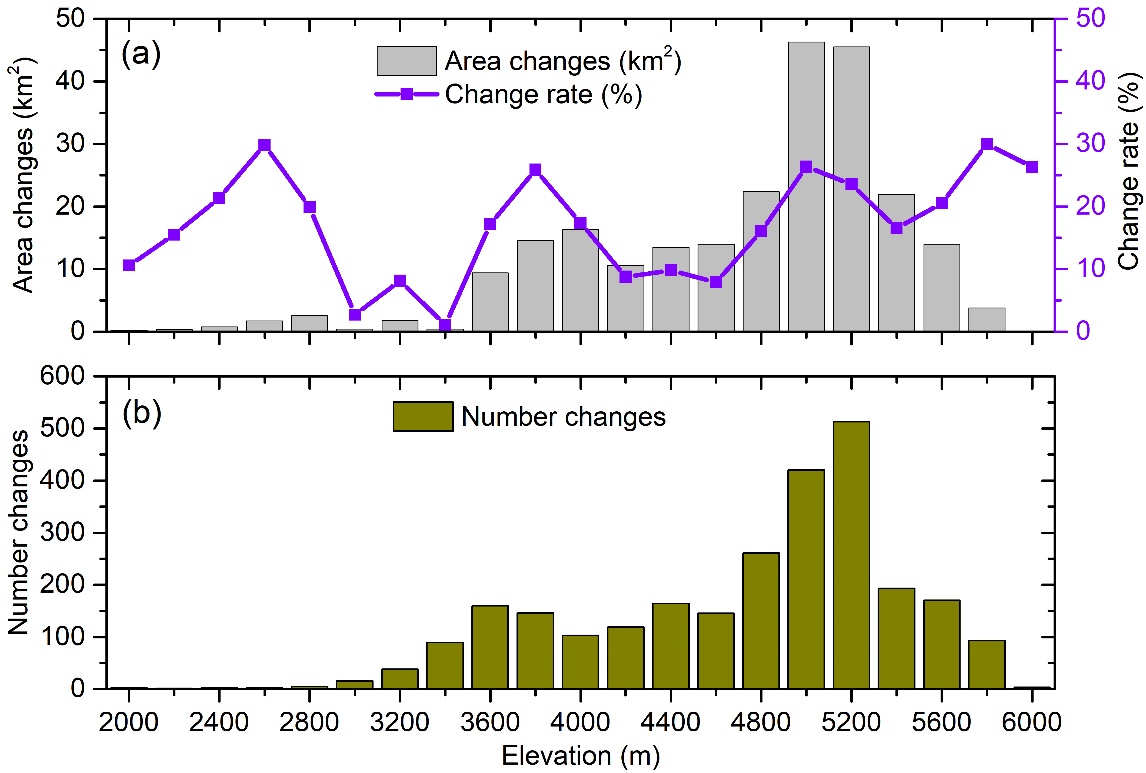


**Figure S1. Changes in the area and number of glacial lakes for each 200 m elevation band in HMA region from 1990 to 2020.** (a) Area changes; (b) Number changes.

**
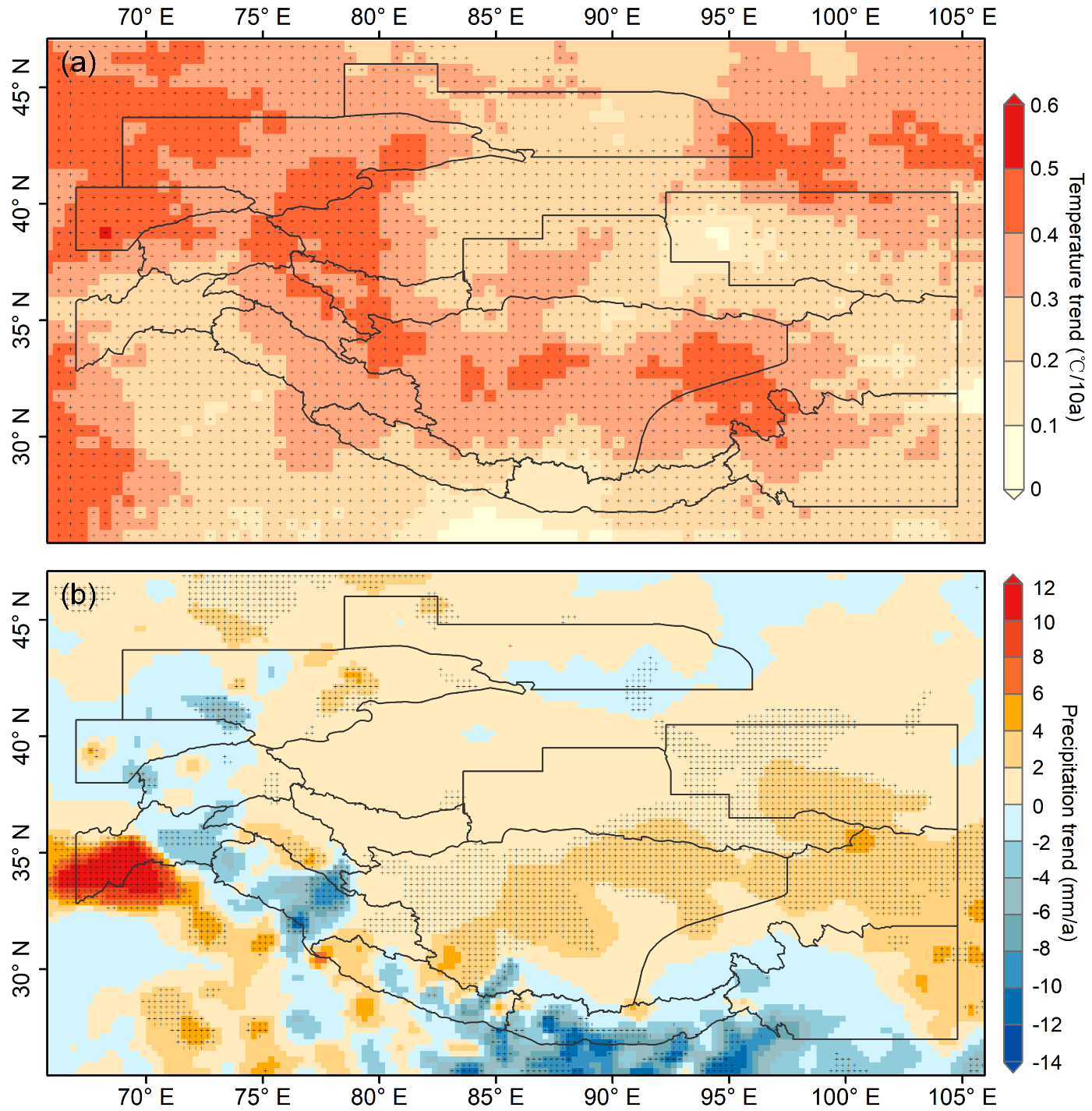
**

**Figure S2. Spatial pattern of temperature and** **precipitation changes in HMA.** (a) Trend in temperature change from 1990 to 2020 produced by CRU data (v4.05, 0.5° spatial resolution); (b) Annual mean precipitation changes for the period 1990–2019 obtained from GPCC data (Full data monthly version 2020, 0.25° spatial resolution). The symbol “+” indicate the linear fit of temperature/ precipitation versus time is at the 90% confidence interval.

**
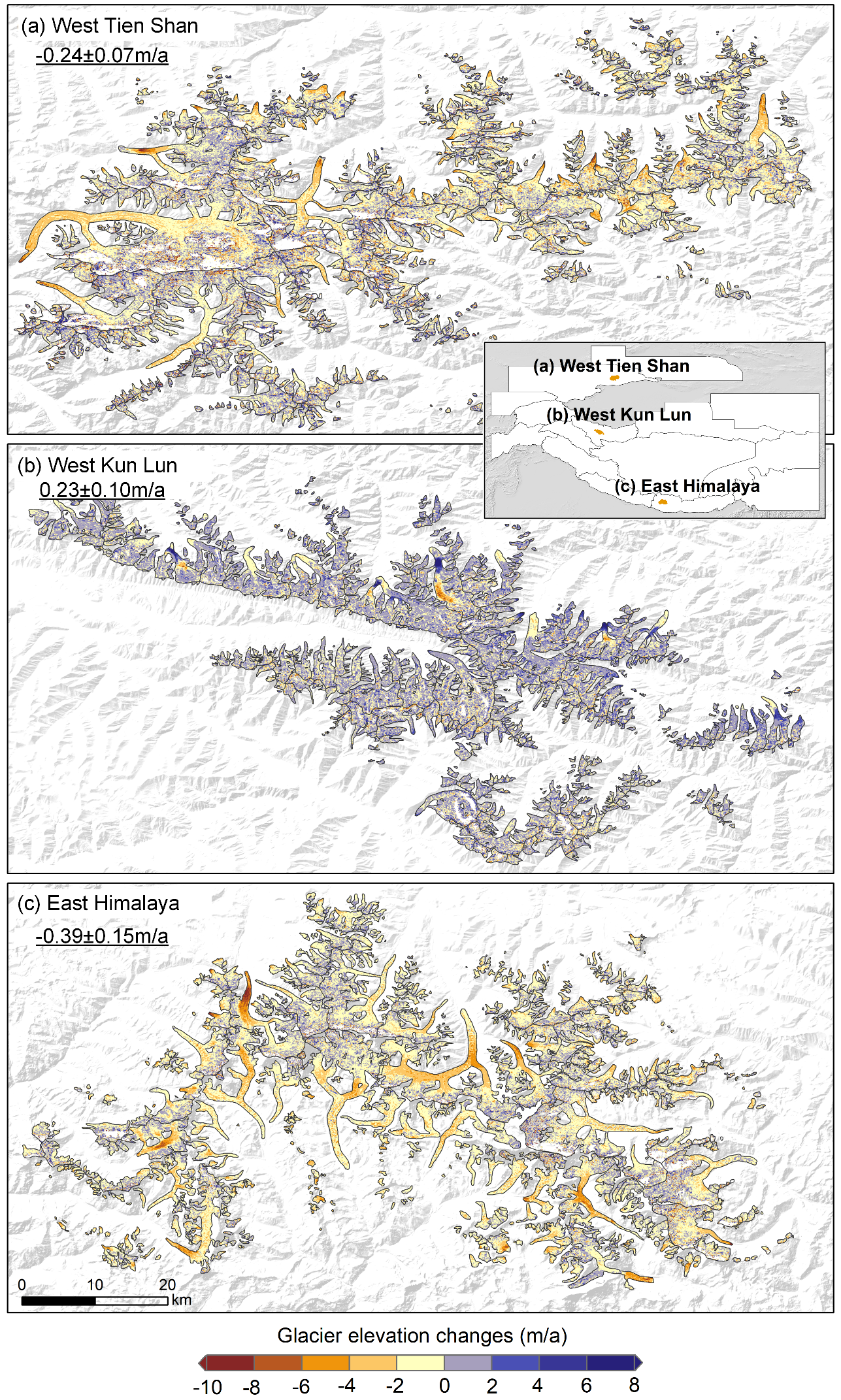
**

**Figure S3. Glacier surface elevation changes in three typical glaciated areas of HMA during 2000–2018.** (a) West Tien Shan; (b) West Kun Lun; (c) East Himalaya. The values in subplots represent the estimated mean glacier elevation changes in each region, respectively.

*
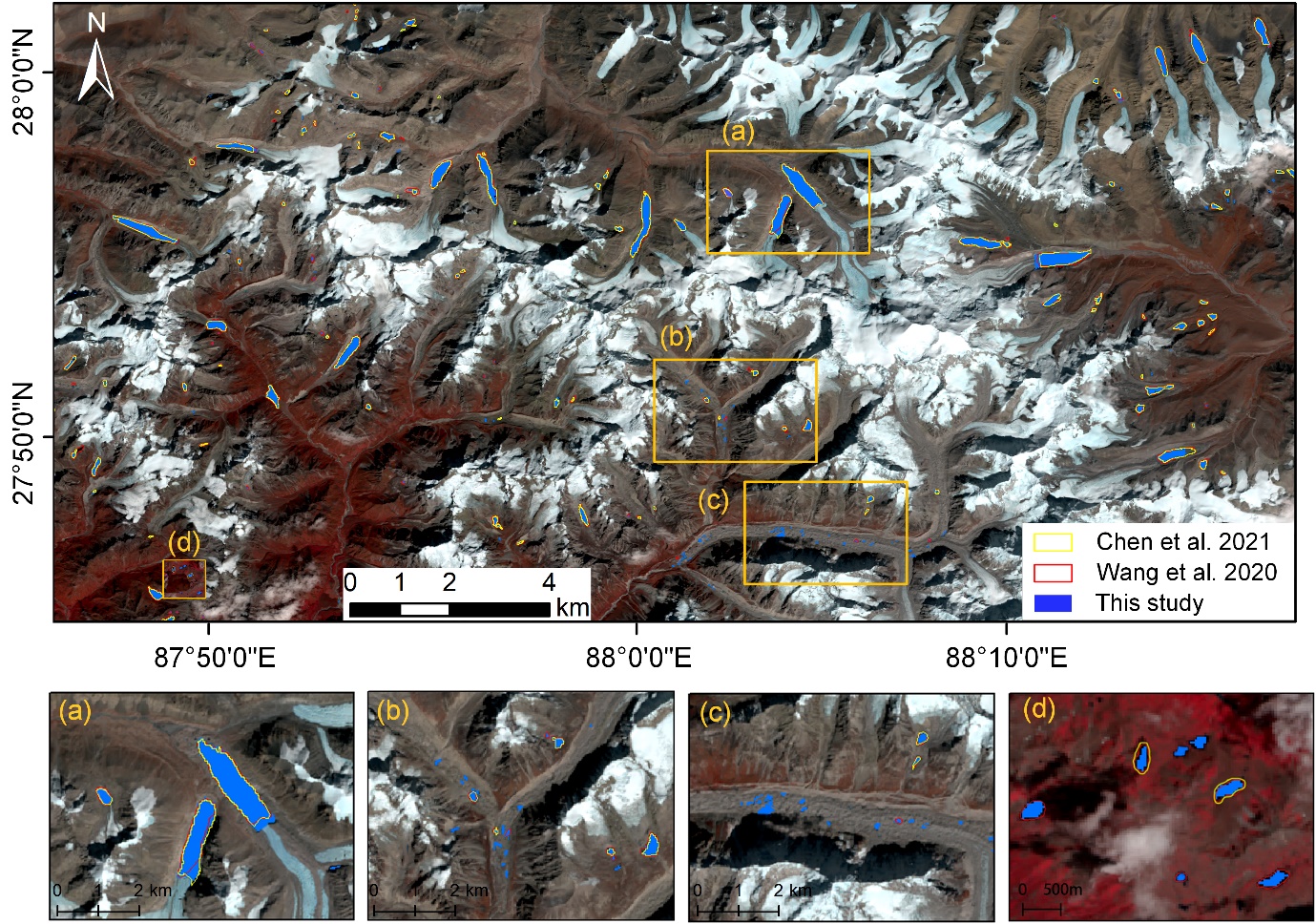
*

**Figure S4. Example showing the mapped glacial lakes by our purposed method, Chen et al. (2021), and Wang et al. (2020) in the Central Himalaya in 2020.** (a)-(d) represent four enlarged maps of glacial lakes. Background image is from false-color composited (bands: 5/4/3) Landsat-8 OLI image in October 2, 2020.


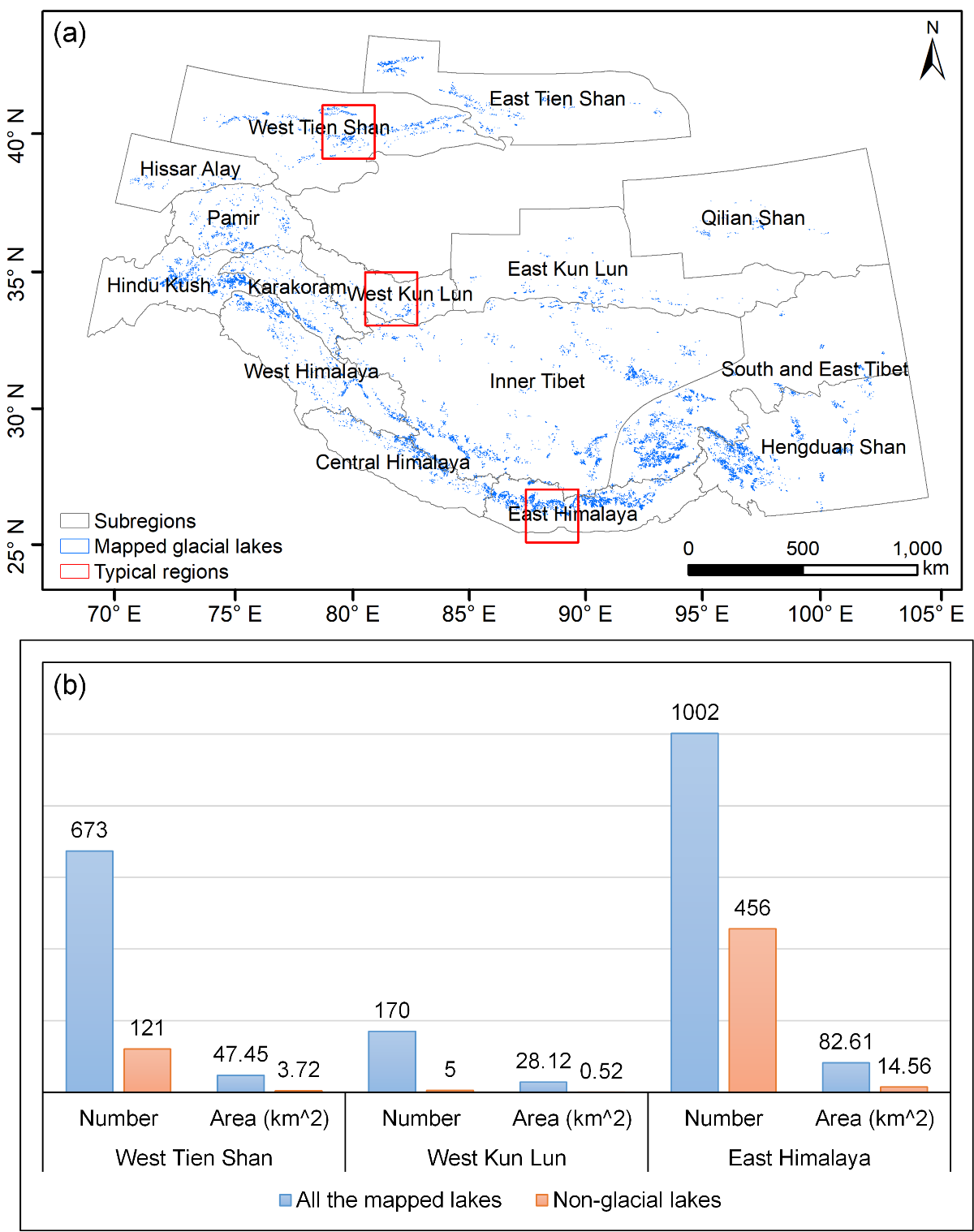


**Figure S5. Examples showing the deviations caused by the non-glacial lake pixels.** (a) Geographic location of three typical regions; (b) Number and area of all the mapped lakes and non-glacial lakes for typical regions in West Tien Shan, West Kun Lun and East Himalaya.

**Table S1**. Comparisons between the two released HMA glacial lake inventories and glacial lakes mapped in this study.

| **Epoch for comparison** | **Source** | **Mapping time** | **Total number** | **Total area (km^2^)** | **Maximum area (km^2^)** | **Minimum area (km^2^)** | **Notes** |
| --- | --- | --- | --- | --- | --- | --- | --- |
| **1990** | Wang et al., 2020 | 1990 | 26089 | 1703.65 | 6.46 | 0.0054 | Altai and Sayan mountains included |
|  |  | 1990 | 19626 | 1453.26 | 6.46 | 0.0081 | Altai and Sayan mountains excluded and areas<0.0081 km^2^ were removed |
|  | Our study | 1990 | 19294 | 1471.85 | 6.35 | 0.0081 | Altai and Sayan mountains excluded |
| **2010** | Chen et al., 2021 | 2010 | 12969 | 1326.66 | 6.42 | 0.0081 | Altai and Sayan mountains excluded |
|  | Our study | 2010 | 21426 | 1634.98 | 6.48 | 0.0081 | Altai and Sayan mountains excluded |
| **2020** | Wang et al., 2020 | 2018 | 28953 | 1955.93 | 6.46 | 0.0054 | Altai and Sayan mountains included |
|  |  | 2018 | 22271 | 1693.80 | 6.46 | 0.0081 | Altai and Sayan mountains excluded and areas<0.0081 km^2^ were removed |
|  | Chen et al., 2021 | 2017 | 15348 | 1395.73 | 6.36 | 0.0081 | Altai and Sayan mountains excluded |
|  | Our study | 2020 | 22646 | 1729.07 | 6.57 | 0.0081 | Altai and Sayan mountains excluded |
